# Potentiometric PVC-Membrane-Based Sensor for Dimethylamine Assessment Using A Molecularly Imprinted Polymer as A Sensory Recognition Element

**DOI:** 10.3390/polym11101695

**Published:** 2019-10-16

**Authors:** Saad S. M. Hassan, Abd El-Galil E. Amr, Heba Abd El-Naby, Mohamed A. Al-Omar, Ayman H. Kamel, Nagy M. Khalifa

**Affiliations:** 1 Chemistry Department, Faculty of Science, Ain Shams University, 11566 Abbasia, Cairo, Egypt; hoba_science@hotmail.com; 2 Pharmaceutical Chemistry Department, Drug Exploration & Development Chair (DEDC), College of Pharmacy, King Saud University, Riyadh 11451, Saudi Arabia; malomar1@ksu.edu.sa (M.A.A.-O.); nkhalifa.c@ksu.edu.sa (N.M.K.); 3 Applied Organic Chemistry Department, National Research Centre, 12622 Dokki, Giza, Egypt

**Keywords:** potentiometric dimethylamine sensor, molecularly imprinted polymers, flow injection analysis, soil samples, method validation

## Abstract

A new simple potentiometric sensor is developed and presented for sensitive and selective monitoring of dimethylamine (DMA). The sensor incorporates a molecularly imprinted polymer, with a pre-defined specific cavity suitable to accommodate DMA. The molecularly imprinted polymer (MIP) particles were dispersed in an aplasticized poly(vinyl chloride) matrix. The MIP is synthesized by using a template molecule (DMA), a functional monomer (acrylamide, AM), cross-linker (ethylene glycol dimethacrylate, EGDMA) and initiating reagent (benzoylperoxide, BPO). Using Trizma buffer solution (5 mmol L^−1^, pH 7.1), the sensor exhibits a rapid, stable and linear response for 1.0 × 10^−5^ to 1.0 × 10^−2^ mol L^−1^ DMA^+^ with a calibration slope of 51.3 ± 0.3 mV decade^−1^, and a detection limit of 4.6 × 10^−6^ mol L^−1^ (0.37 µg mL^−1^). The electrode exhibited a short response time (10 s) and stable potential readings (± 0.5 mV) for more than 2 months. Potentiometric selectivity measurements of the sensor reveal negligible interferences from most common aliphatic and aromatic amines. High concentration levels (100-fold excess) of many inorganic cations do not interfere. The sensor is successfully used for quantification of low levels of DMA down to 0.5 µg mL^–1^. Verification of the presented method was carried out after measuring the detection limit, working linearity range, ruggedness of the method, accuracy, precision, repeatability and reproducibility. Under flow-through conditions, the proposed sensor in its tubular form is prepared and introduced in a two-channel flow injection setup for hydrodynamic determination of DMA. The sampling rate is 50–55 samples h^–1^. The sensor is used to determine DMA in different soil samples with an accuracy range of 97.0–102.8%.

## 1. Introduction

The great needs to control the level of environmental contaminants in natural waterways, potable water, soils, air and biological fluids have generated increasing interest over the recent years [1,2]. Dimethylamine (DMA) as an organic pollutant is a small and simple molecule that is extensively used in many chemical industries as a precursor and raw material. It is used for the production of some surfactant wetting agents, rubber vulcanizing accelerators, accelerators of manufacturing plastic epoxy resins, pigment of polyurethane rubber, leather tanning materials, softeners, lubricants; textile water-proofing agents, cationic surfactants, cellulose acetate rayon treatment substances, rocket propellants, pharmaceuticals (e.g., dimefox, diphenhydramine, metformin, tramadol), agrichemicals (carbamate pesticides, pheromones), and chemical weapons (tabun nerve agent) [3,4]. Dimethylamine (DMA) is also produced by many biological and aquatic systems [5,6,7], associated with some drugs as impurity [8] and released with environmental hazardous waste [9,10].

Some methods and techniques have been suggested for the quantification of dimethylamine and low-molecular weight aliphatic amines. These methods include potentiotitrimetry [11,12,13], spectrophotometry [14,15,16,17,18], spectrofluorometry [19], gas chromatography [20,21,22], high pressure liquid chromatography [23,24], ion chromatography [25,26,27], and capillary electrophoresis [28,29]. Sensors based on color change were also reported in literature. One is based on the use of a pH sensitive dye, which responds through visible color change of bromocresol green indicator to basic volatile dimethylamine [30]. Another is based on the reaction between activated furans and amines [31]. Quartz crystal microbalance electrode coated with chitosan film has also been proposed for the detection of some volatile amines [32]. Most of these methods entailed several time-consuming manipulation steps, involved derivatization reactions; required the availability of sophisticated measuring devices, and use of specific chemicals and reagents.

A cataluminescence (CTL) sensor using ZrO_2_ nanoparticles as the sensing material was introduced for the assessment of low DMA levels in air samples based on the catalytic chemiluminescence (CL) of dimethylamine on the surface of ZrO_2_ nanoparticles at 330 °C [33]. Based on ZnO architectures, a solid-state membrane sensor was prepared and used for DMA determination at 370 °C [34]. Other solid-state sensors based on In_2_O_3_ doped with either MgO loaded with Au, Pd or Pt metal [35], or TiO_2_ loaded with Pt [36] exhibited good sensitivity towards DMA at temperatures > 400 °C. These sensors suffer from serious interfering effect from different anions.

Other commercial solid-state DMA sensors based on conductometric measurements are available in the market [36,37]. However, all the available sensors are operated at high temperature (300–600 °C) and applicable to gaseous form of DMA. In addition, they are expensive (US$ 400–800), revealed a narrow working range (1–20 µg·mL^−1^) and cannot be used to determine DMA with aqueous solutions or at ambient temperatures [33,34,35,36,37].

Potentiometric polymeric membrane sensors are considered as one of the most commonly used techniques for trace analysis of many inorganic, organic and biological analytes at ambient temperature [38,39,40,41,42,43]. The sensing part of most of these sensors consists of suitable electroactive sensing materials dispersed in homogeneous polymeric membranes. Recently, molecularly imprinted polymeric membranes have been introduced as prospective materials for selective recognition of chemical and biochemical sensors [42,43,44,45,46,47,48,49,50,51]. These polymers address one of the most recent advances and challenges relating to sensor technology. Combination of MIPs membranes as receptors with potentiometric sensors have been successfully developed for the assessment of some relatively high molecular mass analytes. However, little is known about the preparation of MIP for small molecules.

In this work, a new cost-effective, ease of manufacturing liquid-contact potentiometric sensor based on artificial host receptors is prepared and used in rapid determination of DMA. The sensing biomimetic receptors are based on the use of acrylamide (AM) as functional monomer. The sensors reveal low detection limit, fast response, good selectivity and recommended application for precise quantification of DMA. Validation of the presented method was carried out after measuring the detection limit, working linearity range, ruggedness of the method, accuracy, precision, repeatability and reproducibility. Under flow-through conditions, the proposed sensors were introduced in a two-channel flow injection setup for continuous monitoring of DMA. The sensors were successfully used in DMA determination in different soil samples under both static and hydrodynamic mode of operations.

## 2. Materials and Methods

### 2.1. Equipment

For all potentiometric measurements, data acquisition (eight-channel electrode-computer interface (Nico-2000 Ltd., London, UK) in connection with a digital mV meter(Orion Model 720/SA, Cambridge, MA, USA) was used. In conjunction with Ag/AgCl double-junction reference electrode (Orion, Model 90-20), the proposed DMA membrane sensors is used as a sensing module. A two-channel peristaltic pump (IsmatechMs-REGLO) and an Omnifit injection valve (Omnifit, Cambridge, UK), was used for the flow injection measurements. An injector with a 100 µL sample loop and polyethylene tubing (0.71 mm i.d.) were utilized.

### 2.2. Reagents

All chemicals were of the highest purity and used as received. Dimethylamine (DMA), sodium tetraphenyl borate (NaTPB), *o*-nitrophenyloctyl ether (*o*-NPOE), high molecular weight poly(vinyl chloride) (PVC), tetrahydrofuran (THF), acrylamide (AM) and ethylene glycol dimethacrylate (EGDMA) were obtained from Fluka (Ronkonoma, city, New York, USA). Benzoyl peroxide (BPO) was purchased from Riedel-deHaen. Acetonitrile, acetic acid, methanol, Tris (hydroxymethyl) amino methane (Trizma) and were purchased from Sigma Chemical Company (St. Louis, MO, USA).

Trizma buffer (5 mmol L^−1^, pH 7.1) was prepared and used as a working buffer solution. A stock solution of 1.0 × 10^–1^ mol L^–1^ DMA^+^ was prepared and the working solutions (1.0 × 10^−2^ to 1.0 × 10^−5^ mol L^−1^) were also prepared by accurate dilutions.

### 2.3. Synthesis of Molecularly Imprinted Polymer

**Figure 1:**
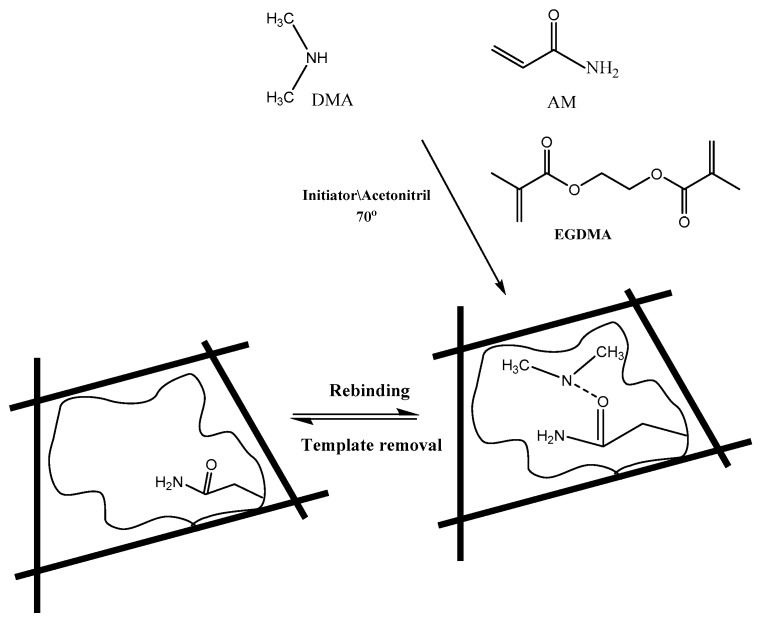
Chemical structure of monomers, cross-linker, and molecularly-imprinted polymers showing non-covalent binding sites to DMA.

The thermal initiated polymerization method was utilized for preparing MIP sensing material. A 0.2 mmol of DMA template and 0.5 mmol of acrylamide (AM) functional monomer were placed in a 25 mL glass tube and dissolved in 15 mL acetonitrile. The solution was exposed to sonication for 1 hour until pre-complex formation between the monomer and template molecules. A 10 mmol of ethylene glycol dimethacrylate (EGDMA) cross-linker and 80 mg of benzoyl peroxide (BPO) initiator were added to the mixture. The mixture was sonicated for 10 min. and then purged with N_2_ gas for 10 min for oxygen removal. The mixture was heated and kept at 80 °C in a water bath for 12 h for complete polymerization. The polymer was collected by vacuum filtration, grinded to a fine powder and thoroughly washed with 100 mL (1:1) methanol/acetic acid several times to ensure complete removal of the un-reacted species. After that, the polymer is washed with methanol and left to dry overnight at room temperature. Similarly, the corresponding non-imprinted polymer (NIP) was synthesized but without the template DMA.A schematic presentation for the imprinting process is shown in Figure 1.

### 2.4. Sensor Construction and Potential (EMF) Measurements

A DMA sensor based on MIP membrane was prepared using a membrane cocktail consisting of MIP (5.2 wt %), tetraphenyl borate (TPB)^-^ (0.9 wt %), PVC (33.0 wt %), and o-NPOE (60.8 wt %). The cocktail was dissolved in 3 mL THF in a Petri-dish with 2.2 cm-diameters. For complete solvent evaporation, the solution of the membrane was left overnight at room temperature to produce a master membrane with 0.1 mm thickness. An Ag/AgCl internal reference wire electrode was placed in 1.0 × 10^−3^ mol L^−1^ of DMA^+^ solution. The sensor was soaked in 1.0 × 10^−2^ mol L^−1^ DMA^+^ for one day for conditioning. When not in use, the sensor was stored in the same solution.

The sensor was calibrated by transferring 10.0 mL of 5.0 × 10^−3^ mol L^−1^ Trizma buffer of pH 7.1to 25 mL beaker. DMA sensor in conjunction with the reference electrode was immersed in the buffer solution. 0.5–1.0 mL aliquots of 1.0 × 10^−5^ to 1.0 × 10^−2^ mol L^−1^ aqueous standard DMA^+^ solution were successively added. The EMF readings were recorded after stabilization to ± 0.2 mV and plotted as a function of the log [DMA]. The plot of the calibration was used for all subsequent measurements of unknown DMA concentration solutions.

### 2.5. Flow-Injection Setup and Continuous Measurements

The flow injection set-up included an Ismatech MSREGLO peristaltic pump in conjunction with an Omnifit injection valve (Rheodyne, 7125, Cambridge, UK). A 200 μL sample loop injector is used for feeding the test sample to a carrier flow consisting of a 5.0 × 10^−3^ mol L^−1^ Trizma buffer (pH 7.1) propelled through polyethylene tubing (0.7 mm i.d.). Dimethylamine potentiometric detector was prepared by the method previously described [45], and inserted with a double junction Ag/AgCl reference electrode downstream. Series of flow runs (three replicate runs) was made on each sample and the average peak height was recorded. This peak height was then compared with a calibration plot made under similar conditions.

### 2.6. DMA Assessment in Soil Samples

A 5.0 g soil sample (finely grounded) was thoroughly treated for 0.5 h with 100 mL Trizma buffer solution (5 mmol L^−1^, pH 7.1) and then filtered. Different aliquots (1.0–10.0 mL) of the filtrate solution were placed in a 25 mL beaker. The DMA electrode was inserted in conjunction with the Ag/AgCl electrode in the test solution and then the EMF was recorded. Spiking technique was also used as mentioned above.

Continuous measurement of DMA in soil samples was conducted by successive injection of 100 µL aliquots of the test solution in a 0.1 mol L^−1^ Trizma buffer (pH 7.1) as a carrier stream. A flow rate of 3.5 mL min^−1^and a single line manifold, in the double-channel pump, was used. The heights of the peaks in terms of mV were recorded and plotted as a function of log [DMA] concentration.

## 3. Results and Discussions

### 3.1. Characterization of the Molecularly Imprinted Polymeric Membrane

**Figure 2:**
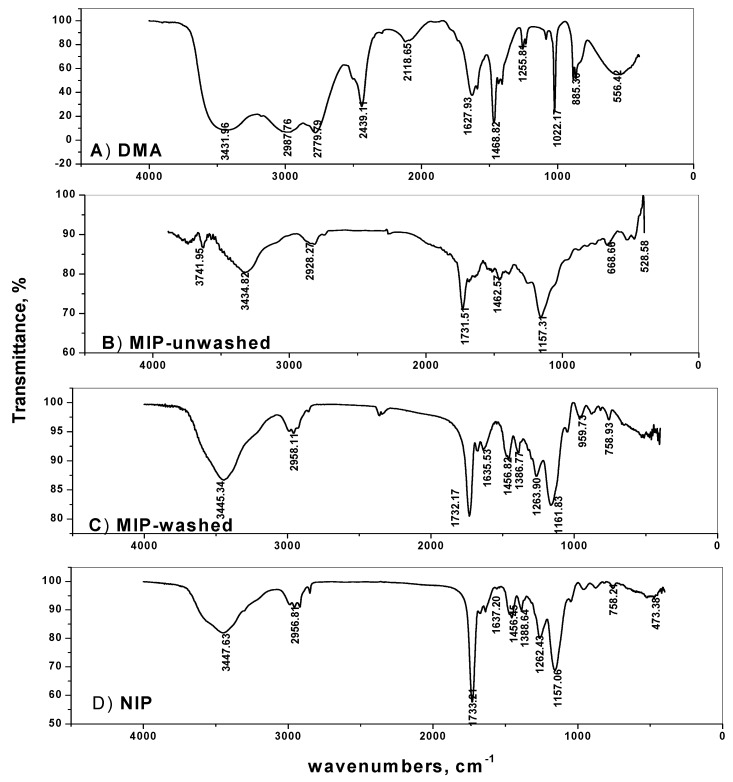
Spectra of: (**A**) DMA, (**B**) MIP-unwashed, (**C**) MIP-washed and (**D**) NIP.

Molecular imprinting using the non-covalent approach was employed for selective recognition of DMA molecules. The (–NH) group in DMA forms strong hydrogen bonding with both the functional monomer used and with the carbonyl group present in the EGDMA cross-linker. The FTIR spectra of MIPs particles confirm the imprinting process through the absence and presence of DMA on the surface of either washed or non-washed polymer particles, respectively (Figure 2). The spectra show two peaks at about 1732 and 1157 cm^−1^, corresponding to –C=O and –C–O stretches, respectively, that are common in all spectra due to the EGDMA cross-linker used. The two peaks at about 2956 and 1456 cm^−1^ are assigned to stretching and bending vibrations of –CH_3_ and –CH_2_– groups present in the cross linker, respectively. The presence of a broad peak at 3447 cm^−1^ assigned to –N–H stretching vibration is due to the amide group of acrylamide monomer. This peak appeared in both NIP and washed MIP particles. In the non-washed MIP particles, the –N–H stretch peak is shifted to 3434.8 cm^−1^ which agree with the –N–H stretch of DMA at 3431.9 cm^−1^. These data reveal that acrylamide monomer was suitably polymerized and DMA was successfully imprinted.

**Figure 3:**
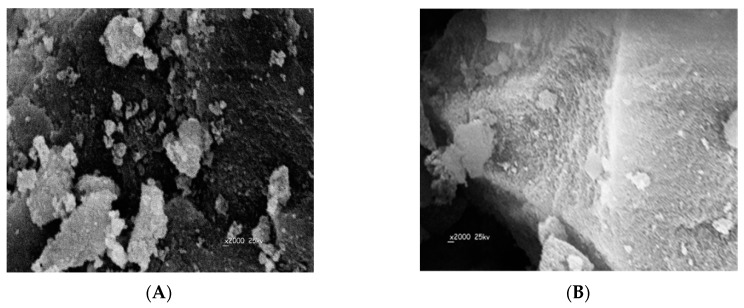
Electron microscopy (SEM) images of: (**A**) MIP and (**B**) NIP.

The surface morphologies of the obtained DMA MIP and NIP particles were characterized by scanning electron microscopy (SEM). As shown in (Figure 3), all MIP particles are of irregular and rough surfaces with an average size of 141.2 µm. This can be attributed to the pores formed during the imprinting process of the template on the polymer. The NIP particles have smoother and uniform shape compared with the MIPs and their average particle size is of 68.5 µm. The difference in particle size between MIPs and NIPs can be attributed to the imprinting effect of DMA.

### 3.2. Response Characteristics of DMA Sensor

**Figure 4:**
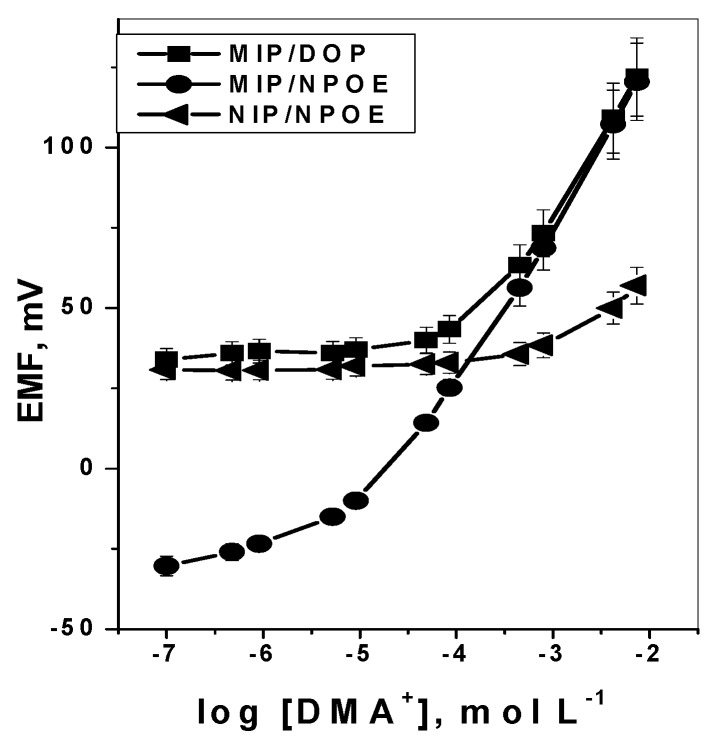
Response curves of: MIPs and NIP membrane based sensors in 5.0 × 10^−^^3^ mol·L^−^^1^ Trizma buffer of pH 7.1.

DMA sensors incorporating membranes with particles of either MIP or NIP dispersed in the PVC membranes were tested and characterized as potentiometric transduction according to IUPAC guidelines [52]. The obtained potential response of a conventional design under static mode of operation is shown in Figure 4. Sensors based on MIP particles exhibited linear potentiometric response towards DMA ion with slope of 48.6 ± 0.3 (r^2^ = 0.999) and 51.3 ± 0.3 (r^2^ = 0.999) mV decade^−1^ and detection limits of 9.0 × 10^−5^ and 4.6 × 10^−6^ mol L^−1^ for membranes plasticized with DOP and *o*-NPOE, respectively. However, sensors consisting of membrane with non-imprinted polymer (NIP) particles show no response towards DMA, probably due to the absence of either DMA receptor cavities or electroactive responsive material in the membrane.

**Figure 5:**
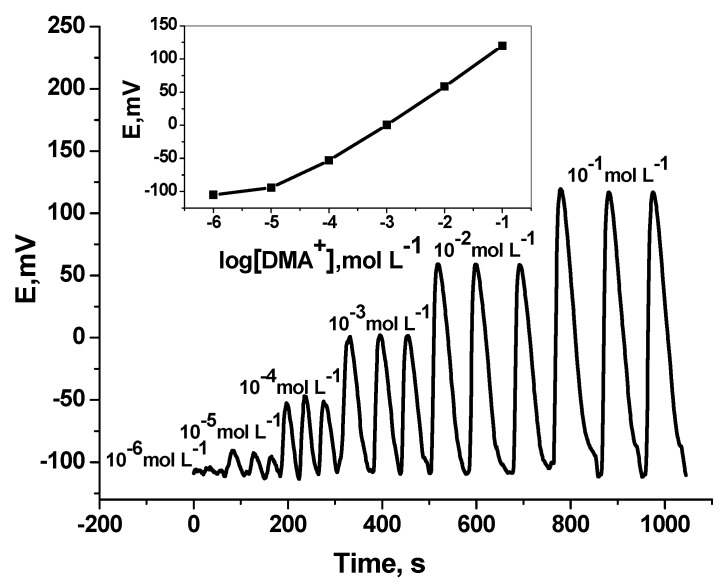
Transient flow injection signals of DMA sensor. Flow conditions: carrier solution, 5.0 mmol·L^−1^ Trizma buffer (pH 7.1); flow rate 3.5 mL min^−1^; loop volume 200 µL.

Under flow-through mode of operation, and using tubular-type detector, measurement of DMA was tested. Over the concentration range 1.0 × 10^−5^ to 10^−2^ mol L^−1^, the sensor revealed sub-Nernstian calibration slope of 50.1 (r^2^ = 0.998) with a detection limit of 1.0 × 10^–5^ mol L^–1^ and sampling rate 50–55 sample h^–1^ (Figure 5) [50]. The results obtained were shown in Table 1.

### 3.3. Method Validation

The quality and consistency of the data obtained by the proposed sensor were confirmed by using the method validation verified by ISO/IEC 17025, AOAC, USP, U.S.EPA, WHO and U.S.FDA [53,54]. To evaluate all method validation parameters such as linearity, detection limit, trueness (accuracy), standard deviation (precision), robustness and selectivity; 6 batches (triplicate each) of standard DMA solution were used.

#### 3.3.1. Linearity and Detection Limit of the Method

The lower DMA detection limit (LOD) under static mode of operation was calculated from the intersection of extrapolated linear segments of the calibration graph according to IUPAC guidelines [52] and found to be 4.6 × 10^−6^ mol L^−1^ (0.37 µg mL^−1^) DMA with a lower quantization limit (LLQ) of 0.71 µg mL^−1^. The linear range of the calibration plots was 1.0 × 10^−2^ to 1.0×10^−5^ mol L^−1^. In the flow injection mode of operation, the method linearity and LOD were 1.0 × 10^−2^ to 8.0 × 10^−5^ mol L^−1^ and 1.0 × 10^−5^ mol L^−1^, respectively.

#### 3.3.2. Method Accuracy and Precision

Precision and accuracy of the proposed method were examined by using six replicate measurements of 10 μg mL^−1^ DMA as an internal quality control sample. Method precision (relative standard deviation, RSD) and accuracy were calculated using:

Accuracy, % = *(x/µ)* × 100 (1)

Precision (RSD), % = *(S/x)* × 100 (2)

Where *x, μ*, and *S* are the average measured analyte concentration, the reference concentration, and standard deviation, respectively (Table 1).

#### 3.3.3. Within-Day Repeatability and Between-Days Reproducibility

The closeness of agreement between mutually independent repetitive test results obtained with 10 µg mL^–1^ internal quality control DMA sample was measured using the proposed sensor and the same reagents during short intervals of time within one working day (within day reproducibility), significantly small variation (± 3%) from the final mV readings was noticed. The RSD was found to be 2.1 and 2.3% for both static and flow-through modes of operation, respectively. Reproducibility of the results (day-to-day response variations) was also tested by measuring 10 µg mL^−1^ internal quality control DMA sample in 5 consecutive days using different batches of the reagents and daily recalibrated. Small variation of the results compared to those obtained for repeatability experiments were obtained. The RSD was found to be 2.8 and 3.1% for both static and flow-through modes of operation, respectively. These data indicate good response stability of the proposed MIP based membrane sensor. With all sensors examined, the detection limits, response times, linear ranges and calibration slopes were reproducible over a period of at least 8 weeks.

#### 3.3.4. Robustness and Ruggedness of the Presented Method

The capacity of the present proposed method to remain un-affected by deliberate change of the method parameters was also tested. A number of method parameters, such as sample pH, sample volume size, flow rate of the carrier solution (in FIA) and the volume of the injected sample were varied within a realistic range, and the effect of the variable parameters is monitored. In addition, two different mV meters and four different DMA sensor assemblies on different days were used for repetitive determination of DMA. Measurements of repeatability (within-day) and reproducibility (between-days) showed variation in potential within the range of 2–3 mV.

The obtained results showed that the effect of these studied parameters lied within the specified tolerance and the variations are considered within the robustness range of the method.

#### 3.3.5. Effect of pH

**Figure 6:**
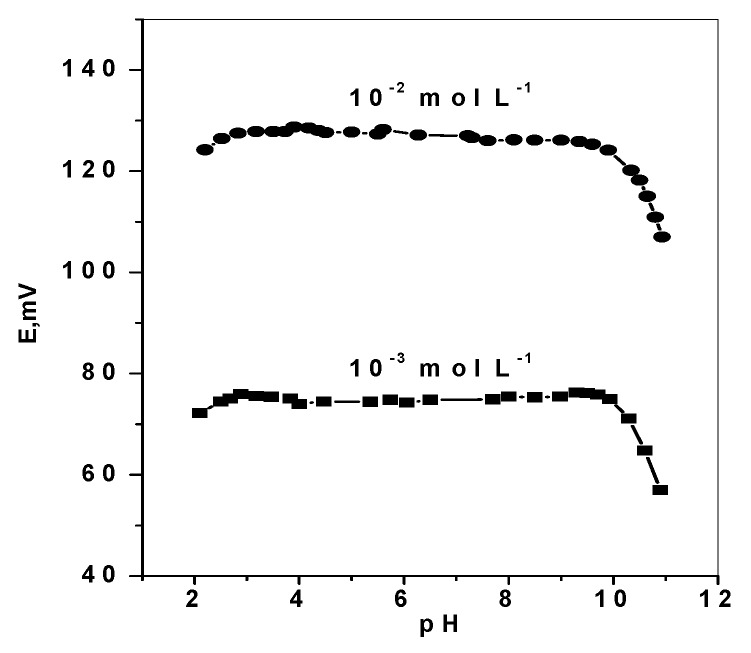
pH effect on the potentiometric response of DMA sensors.

The effect of pH variation on the potentiometric response of DMA based membrane sensors was examined by immersing the sensor in conjunction with a pH electrode and a reference electrode in 10^–3^ and 10^–2^ mol L^–1^ DMA solutions. Dilute sodium hydroxide and /or hydrochloric acid solutions were added to the test solutions and the pH was adjusted to the pH range 2–11. The sensor potential and pH value were simultaneously registered. The pH/potential profile shown in Figure 6, revealed no change in the potential response within the pH range 2.8–9.7. Complete ionization (protonation) of DMA occurred in pH values below its *pKa* (10.7). So, all subsequent measurements using the proposed DMA sensor were carried out using Trizma buffer solution (5 mmol L^−1^, pH 7). Under these conditions, dimethylamineis measured and detected as a monovalent DMA^+^ ion due to complete ionization.

#### 3.3.6. Time Response

The sensor was inserted in different concentrations of DMA in which, each solution has a 10-fold difference. The response time for all DMA concentrations was < 10 s within the linear range of the calibration curves and reflecting the fast response of these proposed sensors. Under flow-through measurements, the response time was 40–60 s as expressed as base line-to- base line response.

#### 3.3.7. Method Selectivity

The extent to which the present proposed DMA membrane sensor can be used to determine DMA in the presence of some inorganic cations, aliphatic and aromatic amines and some amino acids was assessed by measuring the selectivity coefficients ($K_{i,j}^{pot}$) of the sensor. Applying the “fixed interference method (FIM)” [55], the selectivity coefficient values were calculated using Equation (3):

(3) $$K_{i,j}^{pot}=a_{i}/a_{j}^{\left(Z_{i^{}}/Z_{j}\right)}$$

where *a_i_* and *a_j_* are activities and *Z_i_, Z_j_* are the charges of DMA and interfering ion, respectively. In this method, the selectivity coefficients of DMA sensors were evaluated with a fixed concentration of interferent (10^−3^ mol L^−1^) adjusted to pH 7.0 with 5 mmol L^−1^ Trizma buffer. The obtained potential values are plotted versus the activity of the primary ion *a_i_*. The selectivity order for sensors with membranes plasticized with *o*-NPOE was in the order: DMA ˃ Ethylenediamine˃ Histidine ˃ Methylamine ˃ Hexamine ˃ Hydroxylamine > Urea ˃ Aminophenol ˃ Arginin ˃ Alanin (Table 2).

### 3.4. Analytical Applications

It has been reported that soil treated with agricultural dialkyl dithiocarbamate pesticides and herbicides are commonly contaminated with dimethylamine which is released by the degradation of these compounds [56]. Accurate quantification of dimethylamine (DMA) is commonly measured using gas chromatography-mass spectrometry [21]. The method involved a time consuming derivatization reaction and lengthy manipulation steps. Different soil extracts samples were spiked with different standard DMA concentrations. The solutions were buffered at pH 7.1 using 5.0 mmol L^−1^ Trizma buffer solutions. The obtained results showed a mean recovery of 97.0–102.8% as mentioned in Table 3. This reflects a negligible matrix effect. In addition, reasonable accuracy and procedure simplicity are also obtained.

## 4. Conclusions

A simple, reliable and low-cost potentiometric ion-selective electrode for static and hydrodynamic monitoring of dimethylamine is developed and characterized. A newly DMA mimic receptors was synthesized, dispersed in plasticized poly(vinyl chloride) membrane and used as a molecular recognition system. Optimization and verification of the proposed method enable high accuracy, good precision and rapidity in measurements of DMA concentration down to 0.5 µg mL^–1^. The proposed method can be used in DMA determination in different soil samples under batch and flow-through modes of operation. Advantages offered by the proposed sensor are: (i) applicability over a wide range of DMA concentration (0.5–450 µg L^–1^), (ii) fast response at ambient temperature (8 s) (iii) stable potential response (± 0.5 mV), (iv) long life span (>2 months), (v) high accuracy (> 98%), (vi) good precision (< 0.5%), (vii) reasonable selectivity in the presence of many basic organic and inorganic ions, (viii) low fabrication cost (< $10), (ix) application to turbid and colored test solutions and (x) ease of interfacing with automated systems.

## Figures and Tables

**Table 1:** Characteristics of DMA sensors under static and flow-through modes of operation in 5 mmol·L^–1^ Trizma buffer, pH 7.1.

Parameter	Static	Hydro-Dynamic
Slope, (mV decade^–1^)	51.3 ± 0.3	50.1 ± 0.7
Correlation coefficient, (r^2^)	0.9997	0.9976
Linear range, (mol L^–1^)	5.0 × 10^−5^ to 1.0 × 10^−2^	8.0 × 10^−5^ to 1.0 × 10^−2^
Detection limit, (mol L^–1^)	4.6 × 10^−6^	1.0 × 10^−5^
Working range, pH	2.8−9.7	2.8−9.7
Response time, (s)	10	60
Life span, (week)	8	8
Standard deviation, (mV)	0.3	0.7
Accuracy, (%)	99.5	99.1
Precision, CV_w,_(%)	0.5	0.9

**Table 2:** Selectivity coefficients (log $k_{DMA,B}^{pot}$) of DMA membrane sensors plasticized with *o*-NPOE in 5.0 × 10^−3^mol L^−1^ Trizma buffer of pH 7.1.

Interference (*B*)	*Log K^pot^_DMA,J_
Histidine	−2.12 ± 0.5
Alanin	−3.11 ± 0.2
Arginin	−3.07 ± 0.3
Hexamine	−2.52 ± 0.4
Urea	−2.94 ± 0.6
Hydroxylamine	−2.87 ± 0.4
Aminophenol	−3.01 ± 0.3
Methylamine	−2.21±0.5
Ethylenediamine	−2.05±0.3

*Average of 3 measurements

**Table 3:** Assessment in spiked soil extracts samples.

Soil Samples		
Added, mmol L^–1^	Found*, mmol L^–1^	Recovery,%
0.1	0.097 ± 0.006	97.0
1.0	1.028 ± 0.036	102.8
10.0	9.775 ± 0.409	97.8
20.0	19.6 ± 0.2	98.0
